# Commentary: CRISPR-Cas9 mediated editing of starch branching enzyme, SBE2 gene in potato for enhanced resistant starch for health benefits

**DOI:** 10.3389/fgeed.2026.1803282

**Published:** 2026-04-22

**Authors:** Ling Yin

**Affiliations:** 1 College of Life Science, Weifang Institute of Technology, Weifang, Shandong, China; 2 Weill Cornell Medicine, Cornell University, New York, NY, United States

**Keywords:** CRISPR-Cas9, genome editing, potato, resistant starch, starch branching enzyme (SBE2)

## Introduction

The global burden of diet-related metabolic disorders has intensified efforts to improve the nutritional quality of staple crops. Potatoes, a dietary cornerstone for millions, present a particular challenge due to their high glycemic index, primarily attributable to their rapidly digestible starch composition. Enhancing resistant starch (RS) content in potatoes offers a promising avenue for developing healthier food products. In this context, the study by Batta et al. provides a timely and focused investigation into the application of CRISPR-Cas9 genome editing to modify starch biosynthesis in potato, specifically targeting the starch branching enzyme 2 (SBE2) genes (Batta et al., 2025). This commentary critically examines the study’s methodology, situates its findings within the historical and contemporary landscape of starch metabolic engineering, and evaluates its translational significance for developing nutritionally enhanced crops.

## Target selection and technological evolution

The choice of SBE2 as a target is grounded in decades of starch biochemistry research. Starch branching enzymes introduce α-1,6-glycosidic linkages into amylose to form amylopectin, the branched, rapidly digestible component of starch. Inhibiting these enzymes shifts the amylose-to-amylopectin ratio, increasing amylose and, consequently, resistant starch content. Batta et al. build directly upon the foundational work of Schwall et al., who first demonstrated that simultaneous inhibition of SBE A and B isoforms could produce very-high-amylose potato starch (Schwall et al., 2000). The key innovation in the current study lies in the tool employed. While Schwall et al. used antisense inhibition, and later Andersson et al. achieved efficient high-amylose phenotypes using RNA interference (RNAi) (Andersson et al., 2006), Batta et al. deploy CRISPR-Cas9 for multiplexed editing of both SBE2.1 and SBE2.2 genes (Batta et al., 2025). This represents a logical progression in the technological toolkit, moving from transcript suppression to direct, precise genomic modification. The CRISPR-Cas9 approach offers the potential to generate transgene-free edited plants by segregating away the Cas9 construct, a significant regulatory and consumer acceptance advantage over earlier transgenic methods.

## Mechanistic depth and phenotypic validation

A strength of the study is its rigorous connection between genotype and phenotype. The authors confirm successful editing via sequencing and demonstrate significant downregulation of SBE2 gene expression. Most compellingly, they employ ^1^H NMR spectroscopy to quantitatively show a drastic reduction in the degree of starch branching in mutant lines (as low as 1.15%) compared to the wild type (5.46%) (Batta et al., 2025). This biochemical evidence directly links the genetic intervention to the fundamental structural alteration in the starch polymer. This finding resonates with earlier CRISPR work in potato. Tuncel et al. pioneered Cas9-mediated mutagenesis of SBE1 and SBE2, generating a spectrum of starch phenotypes and establishing that strong reduction of both isoforms leads to greatly reduced branching frequency (Tuncel et al., 2019). Batta et al. extend this understanding by focusing on SBE2 in a specific, commercially relevant cultivar.

The study’s phenotypic analysis is further strengthened by integrating insights from recent, cutting-edge research. Zhao et al. pushed the boundary of starch modification by using a DNA-free CRISPR-Cas9 RNP approach to create potato lines with mutations in all alleles of both Sbe1 and Sbe2, resulting in starch essentially devoid of amylopectin (Zhao et al., 2021). Batta et al.'s work, achieving high amylose (up to 95.91%) and resistant starch content through SBE2 editing, can be viewed as capturing a highly beneficial intermediate phenotype. It demonstrates that complete amylopectin elimination (Zhao et al., 2021) is not necessary to achieve a substantial nutritional improvement; significant partial loss-of-function provides a potentially more agronomically viable product with marked health benefits (Batta et al., 2025).

## Structural determinants of nutritional quality

**FIGURE 1:**
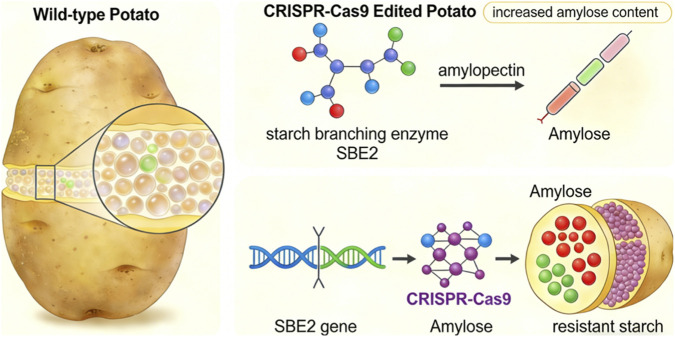
Schematic illustration of CRISPR-Cas9-mediated SBE2 editing and its effect on potato starch composition.

The commentary by Batta et al. wisely frames its structural findings within the established principles of starch digestibility. Through X-ray diffraction (XRD) and scanning electron microscopy (SEM), they show that the edited starches retain the B-type crystallinity typical of tuber starches but exhibit altered granule morphology (Batta et al., 2025). These physical changes are crucial for understanding the enhanced RS content. The work of Martens et al. provides a robust explanatory framework, having identified that amylopectin structure (specifically chain length distribution) and crystalline type are the primary determinants of *in vitro* starch digestion kinetics across botanical sources (Martens et al., 2018). By reducing branching through SBE2 editing, Batta et al. directly alter the amylopectin chain architecture, moving it toward a profile associated with slower digestion. Concurrently, the increased amylose content and altered granule morphology likely reduce the enzymatic accessibility of the starch granules. Thus, the study effectively manipulates the key structural variables that govern nutritional functionality, as defined by Martens et al. (Martens et al., 2018). The mechanistic basis of this manipulation—where SBE2 editing reduces branching activity and shifts starch composition toward higher amylose content—is illustrated schematically in Figure 1.

In wild-type potato, SBE2 (starch branching enzyme 2) introduces α-1,6-glycosidic linkages into amylose to form branched amylopectin, resulting in a starch composition with approximately 20% amylose and 80% amylopectin. CRISPR-Cas9 editing of the SBE2 gene disrupts its function, reducing branching activity and shifting the starch composition toward higher amylose content. Increased amylose content is associated with enhanced resistant starch (RS), which offers nutritional benefits.

Furthermore, the complex interactivity of the starch biosynthetic pathway is acknowledged through the citation of Jayarathna et al. (2024). Their work on stacking mutations in SBE and granule-bound starch synthase (GBSS) genes reveals nuanced interactions that affect final starch granule morphology and molecular order. While Batta et al. focus on SBE2 alone, their success underscores that targeted editing of a single key node can yield dramatic and beneficial outcomes, even within a complex metabolic network.

## Translational context and regulatory considerations

The translational relevance of the study is heightened by its use of “Kufri Chipsona-1”, an elite Indian potato cultivar. This ensures the research addresses regional agricultural and nutritional needs directly. The study’s approach aligns seamlessly with the evolving landscape of crop biotechnology in India, as reviewed by Sharma et al. (2025). These authors highlight genome editing as a powerful tool for addressing food and nutritional security but emphasize the necessity of a clear, science-based regulatory pathway for its products.

A critical aspect requiring further specificity is the regulatory classification of the edited lines. Batta et al. employed CRISPR-Cas9 with the aim of generating transgene-free edited plants, presumably by segregating away the Cas9 construct in subsequent generations. Although the study does not explicitly confirm that the characterized lines are transgene-free, the experimental design—involving segregation of the Cas9 construct—suggests that the edited lines are likely free of transgenic elements, placing them within the SDN-1 category under Indian regulatory guidelines. This distinction is paramount under current regulatory frameworks. In India, the Guidelines for the Safety Assessment of Genome Edited Plants (2022) distinguish between SDN-1 (Site-Directed Nuclease type 1) edits, which involve small insertions/deletions without foreign DNA integration, and SDN-2/SDN-3 edits that may involve template-driven changes. If the Batta et al. lines are confirmed as transgene-free SDN-1 edits, they would likely fall outside the stringent GM regulatory requirements, potentially accelerating their path to commercialization (Sharma et al., 2025). Conversely, if any transgenic elements remain, they would be subject to the more rigorous GM crop approval process. Clarifying this point is essential for accurately assessing the translational potential of this technology.

## Discussion and future perspectives

Batta et al. present a coherent and compelling case study in applied genome editing. They successfully bridge fundamental plant biochemistry with a clear nutritional objective. However, as a proof-of-concept study, it naturally opens avenues for further investigation.

While the study provides strong proof-of-concept data, several limitations warrant discussion. First, the agronomic performance of the edited lines under field conditions remains to be evaluated. Alterations in starch biosynthesis can have pleiotropic effects on plant growth and development. In potato, a tetraploid crop with complex genetics, editing all alleles of SBE2 may lead to unintended consequences, such as reduced tuber yield, altered tuber size distribution, or changes in storage properties. From a practical perspective, any significant reduction in yield or tuber quality would directly impact farmer adoption and commercial viability, regardless of the nutritional benefits. Previous studies have demonstrated that editing starch branching enzyme genes in potato can significantly alter starch composition and granule morphology (Tuncel et al., 2019); however, the agronomic implications of these changes—including potential effects on yield and tuber quality—have yet to be systematically assessed under field conditions. Field trials across multiple seasons and locations are therefore essential to evaluate the stability of the edited phenotype and its impact on overall crop performance.

Second, the processing quality of high-amylose potato starch for industrial applications requires thorough investigation. While resistant starch offers nutritional benefits, it also alters functional properties—such as gelatinization temperature, viscosity, and textural characteristics—that are critical for food processing. For instance, high-amylose starches may exhibit altered swelling power and pasting behavior, which could impact the texture and quality of processed products such as french fries, chips, and reconstituted snacks. These functional changes may require adjustments to processing parameters—such as frying time, temperature, or formulation—to achieve acceptable product quality, underscoring the need for close collaboration between plant breeders and food scientists. The study by Batta et al. demonstrates altered granule morphology, but does not assess how these changes translate to cooking behavior, texture of processed products (e.g., french fries, chips), or storage stability. Sensory analysis of food products made from high-RS potato starch will be critical for consumer acceptance, and collaboration with food scientists and industry partners will be necessary to optimize processing parameters for these novel starches.

Third, the stability of the edited phenotype over vegetative generations is a key consideration for a clonally propagated crop like potato. While CRISPR-induced mutations are generally stable, somaclonal variation during tissue culture and vegetative propagation could potentially lead to phenotypic drift. Because potato is multiplied vegetatively rather than through seed, any instability in the edited trait could be propagated and amplified across generations, potentially compromising product consistency and commercial reliability. Long-term field evaluations over multiple vegetative generations are needed to confirm the stability of the high-amylose trait.

Building on the complex interactions documented by Jayarathna et al. (2024), future research could explore multiplex editing strategies, potentially combining SBE2 edits with modifications to other genes in the starch pathway (e.g., GBSS or starch synthases) to fine-tune functional properties or further enhance RS content. The DNA-free RNP approach demonstrated by Zhao et al. (2021) offers a pathway to generate transgene-free edits without the need for segregation, potentially streamlining regulatory approval.

From a translational perspective, comprehensive field trials are urgently needed to assess yield stability, tuber quality, and performance under diverse environmental conditions. Parallel studies evaluating processing quality—including viscosity profiles, freeze-thaw stability, and sensory attributes—will be critical for industrial adoption. Economic analysis comparing the cost of development and cultivation against the premium for health-enhanced products would provide valuable insights into commercial feasibility.

## Conclusion

In summary, the work by Batta et al. represents a significant step in the continuous refinement of metabolic engineering for crop improvement. By applying precise CRISPR-Cas9 editing to the well-characterized SBE2 target, they achieve a dramatic enhancement of resistant starch in a commercially relevant potato cultivar. The study effectively integrates its findings with the historical trajectory of SBE manipulation, contemporary CRISPR-based advancements, foundational starch biochemistry, and the pertinent regulatory context. It stands as a model for translating fundamental knowledge of plant metabolism into a tangible nutritional trait, demonstrating how precision genome editing can contribute to the development of healthier staple crops for a global population facing escalating dietary health challenges.
